# Bioelectrical impedance analysis of body composition for the anesthetic induction dose of propofol in older patients

**DOI:** 10.1186/s12871-019-0856-x

**Published:** 2019-10-11

**Authors:** Ana M. Araújo, Humberto S. Machado, Amílcar C. Falcão, Patrício Soares-da-Silva

**Affiliations:** 1 Serviço de Anestesiologia, Centro Hospitalar Universitário do Porto, Largo Prof. Abel Salazar, 4099-001 Porto, Portugal; 2 Laboratory of Pharmacology, Faculty of Pharmacy, University of Coimbra, Pólo das Ciências da Saúde, Azinhaga de Santa Comba, 3000-548 Coimbra, Portugal; 3 Department of Biomedicine, Unit of Pharmacology and Therapeutics, Faculty of Medicine, University of Porto, Alameda Prof. Hernâni Monteiro, 4200-319 Porto, Portugal

**Keywords:** Propofol induction dose, Older patients, Phase angle, Frailty

## Abstract

**Background:**

Older people are currently the fastest growing segment of the worldwide population. The present study aimed to estimate propofol dose in older patients based on size descriptors measured by bioelectrical impedance analysis (BIA).

**Methods:**

A cross sectional study in adult and older patients with body mass index equal to or lower than 35 kg/m^2^ was carried out. BIA and Clinical Frail Scale scoring were performed during pre-operative evaluation. Propofol infusion was started at 2000 mg/h until loss of consciousness (LOC) which was defined by “loss of eye-lash reflex” and “loss of response to name calling”. Total dose of propofol at LOC was recorded. Propofol plasma concentration was measured using gas chromatography/ion trap-mass spectrometry.

**Results:**

Forty patients were enrolled in the study. Total propofol dose required to LOC was lower in Age ≥ 65 group and a higher plasma propofol concentration was measured in this group. 60% of old patients were classified as “apparently vulnerable” or “frail” and narrow phase angle values were associated with increasing vulnerability scores. In the Age ≥ 65 group, the correlation analysis showed that the relationship between propofol dose and total body weight (TBW) scaled by the corresponding phase angle value is stronger than the correlation between propofol dose and TBW or fat free mass (FFM).

**Conclusions:**

This study demonstrates that weight-based reduction of propofol is suitable in older patients; however FFM was not seen to be more effective than TBW to predict the propofol induction dose in these patients. Guiding propofol induction dose according to baseline frailty score should also be considered to estimate individualized dosage profiles. Determination of phase angle value appears to be an easy and reliable tool to assess frailty in older patients.

**Trial registration:**

ClinicalTrials.gov Identifier: NCT02713698. Registered on 23 February 2016.

## Background

Older people are currently the fastest growing segment of the worldwide population, and older patients are increasingly submitted to a multiplicity of surgical procedures [1, 2]. Physiologic changes and cognitive dysfunction associated with age, the presence of multiple comorbidities, the use of multiple medications and frailty increase the risk of older patients to adverse postoperative outcomes [1]. As a result, older people present a larger variation in terms of morbidity and physiological characteristics, which affect the predictability of their response to medications [3]. Variation in frailty likely contributes to this heterogeneity but its effects on pharmacokinetics (PK) and pharmacodynamics (PD) are not yet well understood [3]. Consequently, chronological age is a weak contributing factor of pharmacological response. Age-related physiologic changes per se are not always clinically significant and should not result in the same dose regimen alterations for all chronological old patients [3]. Despite these considerations, current guidelines recommend lower doses of anesthetic agents for all chronologically older patients [4–6]. For induction of general anesthesia with propofol, it is generally recommended to decrease propofol dose from 40 mg every 10 s (2–2.5 mg/kg) to 20 mg every 10 s (1–1.5 mg/kg) until induction onset [7].

Additionally, bolus dose recommendations for adult non-obese patients are generally made as a function of total body weight (TBW) [8]. Physiologic changes that occur with aging, such as a decrease in total body water, a decrease in lean body weight (LBW) and an increase in body fat, may possibly require the adjustment of propofol doses based on different weight scalars [5, 9]. Mitchell et al. [9] have advocated that LBW may be an alternative choice for drug dose calculation in older patients. Nevertheless, the adequacy of TBW or other size descriptors as predictors of the propofol dose at LOC has not been studied yet in older patients.

Bioelectrical impedance analysis (BIA) is a reference method for the assessment of body composition by measuring resistance and reactance [9, 10]. Resistance is associated with the quantity of water present in tissues, and reactance is the resistive effect created by the cell membranes and tissue interfaces [11]. Phase angle can be calculated from resistance and reactance [arc-tangent (Reactance/Resistance)*180°/π] and it characterizes the relative effects of fluid (resistance) and cellular membranes (reactance) of the body [11, 12]. Consequently, phase angle has been interpreted as a determining factor of body cell mass, cell membrane integrity, intra and extracellular water distribution and it has also been proposed as a biological sign of cell death [10, 13]. The phase angle varies with sex, age and body mass index (BMI) but the average value for a healthy individual is generally comprised between 6 and 9 degrees [14].

Phase angle evaluation has been suggested as a prognostic factor in a number of clinical disorders, namely, human immunodeficiency virus infection, liver cirrhosis, chronic obstructive pulmonary disease, hemodialysis, sepsis and lung, breast, colorectal and pancreatic cancer [10]. BIA derived phase angle has also been proposed as an objective measure to recognize frail patients [15]. More recently, Mullie et al. [12] concluded that phase angle is strongly associated with sarcopenia and frailty in older patients, and that it is a promising noninvasive device to assess frailty.

Given the physiologic changes in body composition associated with aging, we planned to determine the appropriate size descriptor, measured by bioelectrical impedance, to estimate propofol induction dose in older patients. We also proposed to assess changes in phase angle values in frail patients. We hypothesized that LBW would be a more appropriate dosing scalar to calculate propofol induction dose in older patients and that old frail patients would require lower doses of propofol during induction of general anesthesia.

## Methods

This study was performed at Centro Hospitalar e Universitário do Porto, Porto, Portugal, after Hospital Review Board and Ethical Committee approvals (IRB: N/REF.ª 2015.221(183-DEFI/165-CES) and it provides secondary analyses of data registered at clinicaltrials.gov under the reference NCT02713698 on 23 February 2016.

The main purpose of the research published in clinical trials consisted in the development of a population pharmacokinetic-pharmacodynamic model for propofol when used for induction and maintenance of general anesthesia. Secondarily, sectorial evaluations of the results focusing on the induction and maintenance phases of anesthesia have also been conducted. The first study, planned to assess the ability of body size descriptors to estimate propofol induction dose in obese patients, has already been published [16].

The proposed study is a cross sectional analytical study and it conformed to the requirements of STROBE (Strengthening the reporting of Observational studies in epidemiology) statement. The whole anesthetic procedure was standard except for additional body composition assessment with body composition monitor (BCM, Fresenius Medical Care, Germany) and arterial blood sample. Written informed consent was obtained from all subjects participating in the trial.

### Patients

Adult patients (18–64 years), with ASA (American Society of Anesthesiology) physical status I to III and BMI equal to or lower than 35 kg/m^2^ scheduled for elective nose, ear or general surgery and older patients (with 65 or more years), with ASA physical status I to III and BMI equal to or lower than 35 kg/m^2^ scheduled for orthopedic or elective nose or ear surgery were included in the study.

Exclusion criteria included the presence of predictive criteria for difficult airway management, dementia, severe hepatic or renal insufficiency, significant hemodynamic instability prior to the surgery and a known allergy to propofol at the time of enrolment. Patients with a pacemaker and pregnant women were also excluded.

### Analysis of body composition by bioelectrical impedance

BIA was performed by Body Composition Monitor (BCM, Fresenius Medical Care, Germany) during preoperative evaluation in accordance with the manufacturer’s recommendations [17].

Phase angle values at 50 kHz were registered and standardized values (z-score) were calculated based on reference values generated in a healthy German population [18] according to sex, age and BMI.

The values of resistance and reactance obtained at 50 kHz were used to estimate fat free mass (FFM) or fat mass (FM) based on specific predictive equations [19]. The equation proposed by Kyle et al. [20] was used in adult patients and the equation proposed by Roubenoff et al. [21] was used in older patients for the assessment of FFM.

$$ FFM\ \left( Kyle\ equation\right)=-4.104+\frac{\mathrm{0,518}{Height}^2}{Resistance}+0.231\times TBW+0.130\times Reactance+4.229\times Sex $$

$$ FFM\ \left( Roubenoff\ equation\right)=-5.741+\frac{0,4551{Height}^2}{Resistance}+0.1405\times TBW+0.0573\times Reactance+6.2467\times Sex $$

### Frailty assessment tools

The 9-point Clinical Frailty Scale (CFS) [22] developed by Geriatric Medicine Research (Dalhousie University, Halifax, Canada) was applied to each patient during preoperative assessment. The CFS was assigned from 1 (very fit) to 9 (terminally ill).

### Anesthetic procedure

All patients were brought to the operating room without premedication. In the operating room, continuous pulse oximetry, electrocardiography, invasive blood pressure and neuromuscular blockade were applied in all patients. The bispectral index (BIS) was monitored using a BIS VISTA™ Bilateral Monitoring System (Covidien, Colorado, US) with a bilateral sensor on the forehead of the patient.

Each subject received a standardized dose of fentanyl (2 μg/kg) or a target controlled infusion of remifentanil (pharmacokinetic model of Minto [23]) was set at 3 ng/mL before propofol infusion. Propofol infusion (using Orchestra™ Mobile stand, Fresenius Vial, Brézins, France) was started at 2000 mg/h (33.3 mg/min) until LOC, defined by “loss of eye-lash reflex” and “loss of response to name calling”. Total dose of propofol at LOC was recorded in all patients. After LOC, rocuronium was administered, propofol infusion rate was guided by the BIS and volume-controlled ventilation was started in all patients. Ventilation parameters setting were: tidal volume 7 ml/kg, respiratory rate 12–14 cycles per minute to attain normocapnia and PEEP 5cmH_2_O.

### Quantification of propofol concentration

Arterial blood samples were collected after LOC and they were centrifuged (2862×g for 5 min) at the end of the surgery. Serum samples were then preserved at − 80 °C until analysis.

Propofol concentration was determined by gas chromatography/ion trap-mass spectrometry (GC/IT-MS) using the method described by Campos et al. [24]. The concentration range for the calibration curve was defined according to the expected serum concentrations (0.25, 0.5, 1, 2, 4, 5 and 10 μg/mL).

### Sample size

Literature shows a moderate relationship (*r*^2^ = 0.49) between propofol induction dose and TBW in adult patients with normal BMI [8]. To the author’s knowledge there is no previous study evaluating the relationship between TBW and propofol induction dose in older patients. As a result, sample size considerations were based on association analyses using the Pearson correlation test. To detect a correlation of at least 0.7 between propofol induction dose and TBW a sample of at least 13 subjects was calculated to provide 80% power and a 0.05 level of significance.

### Statistical methodology

The Shapiro-Wilk test was used to test for the normality of data. Categorical variables are presented as frequency (%). Continuous data are presented as mean or median (coefficient of variation, CV %). For comparison between groups, the Student’s t-test and the Mann-Whitney-Wilcoxon test were used for continuous variables. The Fisher’s exact test was used for categorical variables.

Fisher’s exact test was used to estimate the association between CFS and phase angle values: *p*-values, odds ratio (OR) and respective 95% confidence intervals were obtained.

The relation among propofol dose at LOC, body size descriptors and phase angle was determined by linear regression analysis.

Statistical analyses were performed using GraphPad Prism 8.2.0 software (GraphPad Prism, San Diego, California). A *p*-value < 0.05 was considered to be statistically significant.

## Results

Twenty adult patients (Age < 65 group) and twenty older patients (Age ≥ 65 group) were enrolled for participation in the study from April 2016 to March 2017. Patient’s demographics and comorbidity indexes are presented in Table 1.

**Table 1:** Patient demographic characteristics and comorbidity indexes

Variables	Age < 65 group	Age ≥ 65 group
Age (years)	42.85 (26)^a^	77.8 (11)^a^
Gender		
Female	14 (70)^b^	12 (60)^b^
Male	6 (30)^b^	8 (40)^b^
ASA		
I	7 (35)^b^	1 (5)^b^
II	12 (60)^b^	15 (75)^b^
III	1 (5)^b^	4 (20)^b^
Charlson Comorbidity index		
0–1	18 (90)^b^	13 (65)^b^
2–3	2 (10)^b^	6 (30)^b^
4–5	0	1 (5)^b^

*ASA* American society of anesthesiology

^a^Data are present as mean (CV %)

^b^Data are presented as frequency (%)

**Fig. 1:**
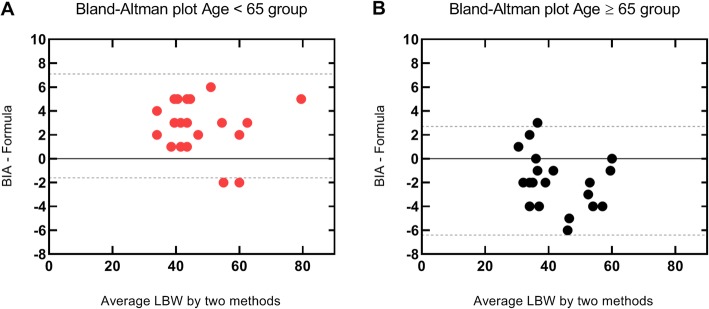
Bland–Altman plots - Janmahasatian formula vs. body impedance analysis in Age < 65 group (**a**) and Age ≥ 65 group (**b**). The 95% limits of agreement are shown as dashed lines. BMI | Body mass index (kg/m^2^)

The body composition of each patient evaluated by BIA is demonstrated in Table 2. The mean percentage of FFM in Age < 65 and Age ≥ 65 groups is 33.5 and 30.2%, respectively. There is a good agreement between the LBW values obtained by the Janmahasatian equation (LBW_j_) [25] and FFM values measured by the BCM in both groups (Fig. 1).

**Table 2:** Body composition according to bioelectrical impedance analysis

Variables	Age < 65 group	Age ≥ 65 group	*p*-value
Body mass index (kg/m^2^)	25.9 (15)	24.3 (14)	0.09
Total body weight (kg)	70.2 (21)	63.0 (18)	0.18
Fat free mass (kg)	49.0 (23)	41.9 (23)	0.04
Fat free mass (%)	70.0 (10)	66.2 (10)	0.09
Fat mass (kg)	21.1 (33)	21.1 (24)	0.97

Data are present as mean (CV%)

**Fig. 2:**
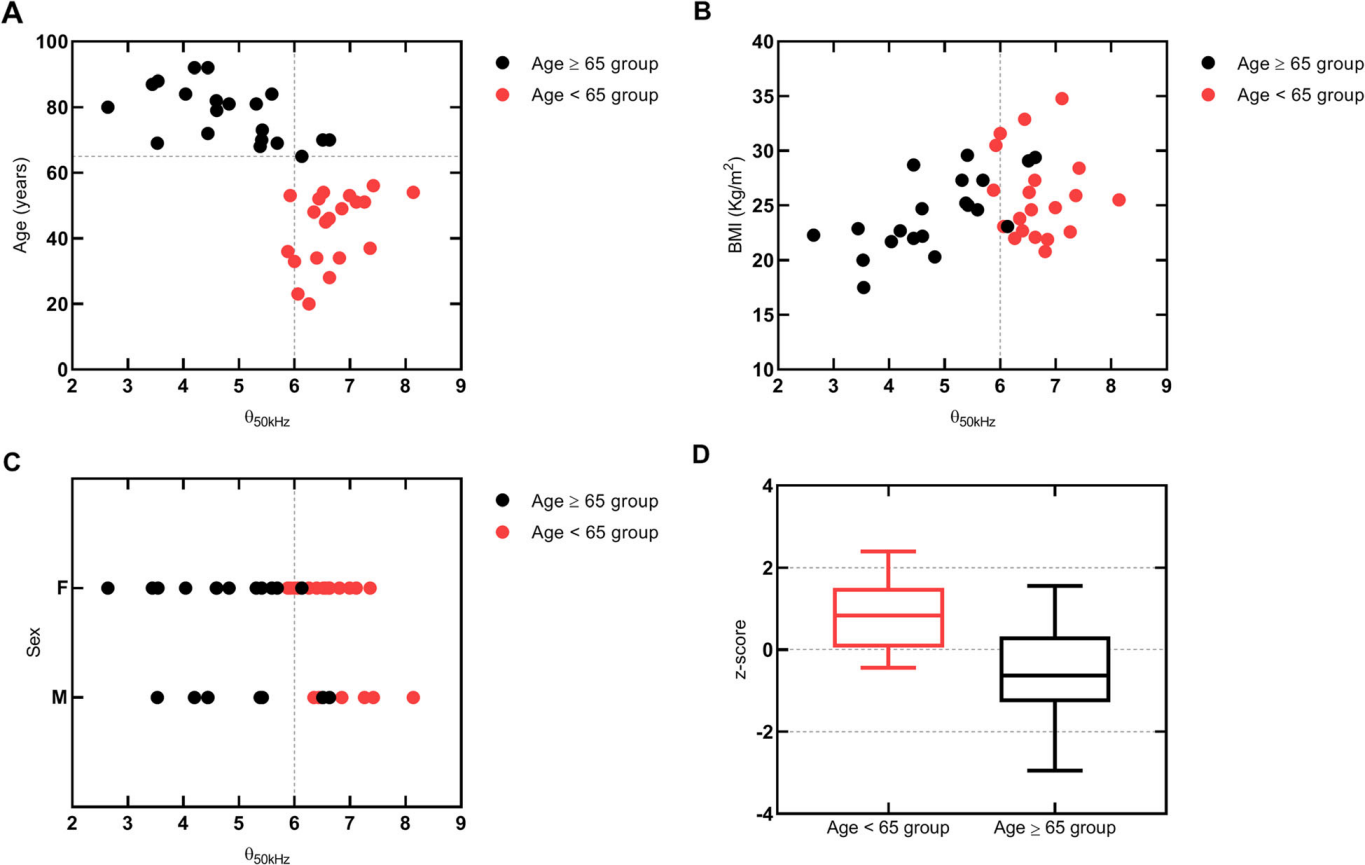
Phase angle reference values. **a**, **b** and **c** represent the distribution of phase angle values according to age, BMI and sex. Vertical dashed lines represent lower phase angle reference value in healthy adult population. **d** shows the distribution of the standardized phase angle, *z score*, [*z score* = (observed phase angle-mean reference phase angle)/SD reference phase angle] for specific age, sex and BMI categories. BMI | Body mass index (kg/m^2^). F | Female. M | Male. SD | Standard deviation

With respect to phase angle values, Fig. 2 shows that lower values were observed in Age ≥ 65 group in comparison with those observed in the Age < 65 group. Figure 2 also shows that in Age ≥ 65 group the median standardized phase angle value *(z-score)* was lower than expected from a reference healthy population with the same sex, age and BMI.

No relevant changes in heart rate, blood pressure and BIS values were observed following opioid administration.

Table 3 shows that the median absolute dose of propofol to LOC was lower in the Age ≥ 65 group (*p* = 0.005). Despite the lower propofol induction dose, the mean variation of MAP (mean arterial pressure) was higher in the Age ≥ 65 group (*p* = 0.046). In this group, 6 patients showed a variation of MAP values before and after propofol dose higher than 40%. These patients were treated with ephedrine and fluid boluses to return MAP to baseline levels. No changes in heart rate that have required intervention were observed during propofol induction. Table 3 also shows that mean propofol plasma concentration after induction of general anesthesia was higher in the Age ≥ 65 group.

**Table 3:** Propofol dose until loss of consciousness

Variables	Age < 65 group	Age ≥ 65 group	*p*-value
Propofol (mg)	90 (40)^a^	68.5 (36)^a^	0.005
Propofol (mg/kg of TBW)	1.24 (30)^a^	1.09 (25)^a^	0.05
Time to LOC (min)	2.95 (1.18)^b^	2.17 (0.78)^b^	0.02
MAP variation^d^ (mmHg)	19.62 (67)^c^	29.04 (54)^c^	0.05
Plasma [Propofol] (μg/mL)	3.96 (39)^c^	7.68 (34)^c^	< 0.001
Time after LOC^e^ (min)	1.6 (1.3)^b^	1.5 (1)^b^	0.84
BIS^f^	49.5 (22)^a^	46 (27)^a^	0.88

*LOC* Loss of consciousness, *MAP* Mean arterial pressure, *TBW* Total body weight

^a^Data are presented as median (CV%)

^b^Data are presented as mean (SD)

^c^Data are presented as mean (CV%)

^d^Variation = [(MAP before propofol – MAP after propofol)/MAP before propofol]*100

^e^Time after LOC is the time when arterial blood sample was obtained after LOC

^f^Bispectral index value at the time of blood sample collection

**Fig. 3:**
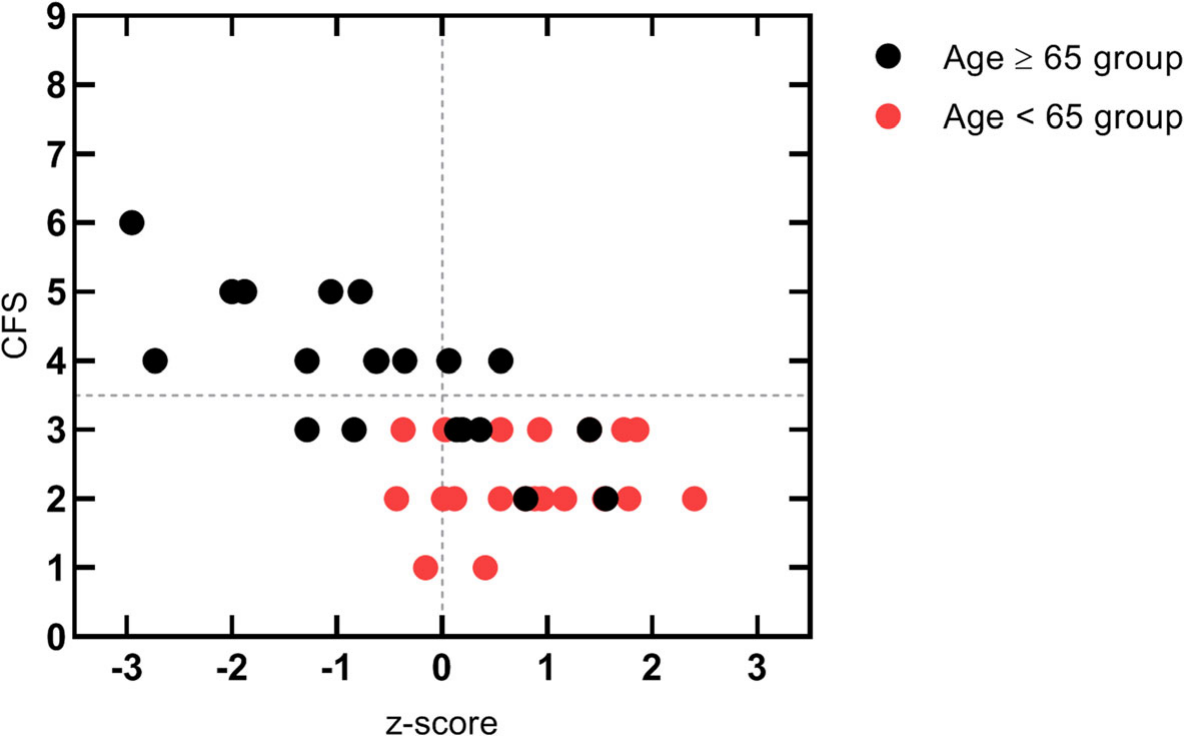
Standardized phase angle *(z-score)* and clinical frail scale scores for each patient. CFS | Clinical frail scale

Clinical frailty scale scores are presented in Table 4. These scores are statistically significant different between groups (*p* < 0.001). Actually, in the Age ≥ 65 group, 60% of patients were classified as *apparently vulnerable* or *frail* and in the Age < 65 group all the patients were classified as *very fit*, *well* or *managing well*. Figure 3 shows that lower z-scores are associated with high vulnerability CFS scores. The patients with a CFS score equal to or higher than 4 have been 23 times more often classified with a z-score lower than zero than patients with a CFS score lower than 4 (OR 0.044; 95% confidence interval 0.0072 to 0.2630; *p* = 0.0002).

**Table 4:** Clinical frailty scale

Age < 65 group	Clinical frailty scale		Age ≥ 65 group
	Item	Description	
2 (10)^a^	1	Very fit	0
11 (55)^a^	2	Well	2 (10)^a^
7 (35)^a^	3	Managing well	6 (30^)a^
0	4	Apparently vulnerable	7 (35)^a^
0	5	Mildly frail	4 (20)^a^
0	6	Moderately frail	1 (5)^a^
0	7	Severely frail	0
0	8	Very severely frail	0
0	9	Terminally ill	0

^a^Data are presented as frequency (%)

**Fig. 4:**
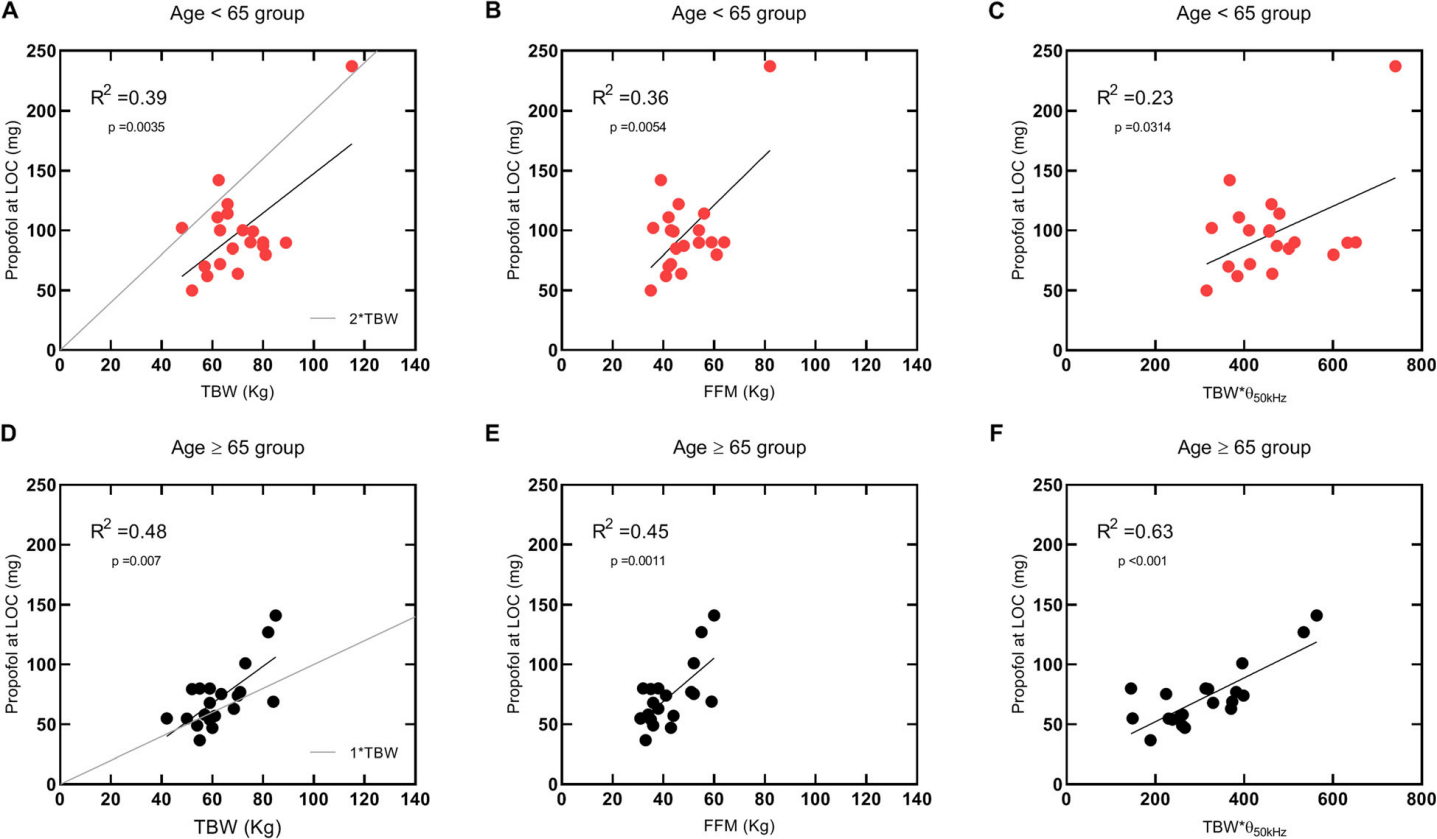
Propofol dose at LOC. **a**, **b** and **c** show, respectively, the correlations of propofol induction dose with TBW, FFM and TBW•phase angle of Age < 65 group. **d**, **e** and **f** illustrate, respectively, the correlations of propofol induction dose with TBW, FFM and TBW•phase angle of Age ≥ 65 group. Grey line in **a** and **d** represents the function equivalent to the standard adult and older propofol dose (2 mg•TBW in **a** and 1mg•TBW in **d**), respectively. BMI | Body mass index. FFM | Fat free mass. LOC | Loss of consciousness. TBW | Total body weight

Regarding the regression analysis, Fig. 4 indicates that propofol dose was similarly related to TBW and FFM in both groups (*r*^2^ = 0.39 vs 0.36 and *r*^2^ = 0.48 vs 0.45). When the TBW is scaled by the corresponding phase angle values, Age ≥ 65 group shows a stronger relationship than that observed in the Age < 65 group (*r*^2^ = 0.63 vs 0.23). Figure 4 also compares the propofol doses at LOC with those that would be obtained using a standard bolus approach. Accordingly, the use of a bolus dose of propofol based on TBW (1 mg•TBW) would result in inappropriately low doses in some patients of the Age ≥ 65 group (Fig. 4d). Conversely, the use of the standard dose (2 mg•TBW) would be adequate for the Age < 65 group (Fig. 4a).

## Discussion

In this study, older patients required a lower propofol dose than adult patients to clinical LOC when propofol was administered at a fixed infusion rate. In the Age ≥ 65 group, the relationship between propofol dose and TBW scaled by the corresponding phase angle values is stronger than the correlation between propofol dose and TBW or FFM.

In the present study, LOC was defined clinically by “loss of eye-lash reflex” and “loss of response to name calling”. BIS values were not used to guide propofol dose because of the previously reported delay between clinical recognition of LOC and the decrease in BIS values [26], which may lead to propofol overdosing. The same infusion rate of propofol was adopted in the Age < 65 and Age ≥ 65 groups because propofol infusion rate has a critical impact on the dose and time required to induction of general anesthesia [27–29]. The use of different infusion rates appear alone to result in different dosing regimens and correlations levels. Additionally, a lower infusion rate (2000 mg/h) than that exposed in the recommendations (7200 mg/h) have been selected. According to Chan et al. [30] the use of slower infusion rates allowed more time to observe the specific clinical endpoint of LOC. It has also been published in several studies that the mean MAP variations were lower when slower propofol infusion rates (2000–3000 mg/h) were used [30–32].

For the fixed infusion rate adopted in the present study, propofol dose to LOC in adult patients was similarly correlated with TBW and FFM, as also noticed by Ingrand et al. [8]. Similar considerations were herein observed in the results obtained for the Age ≥ 65 group (Fig. 4). These results are in accordance to the results of body composition evaluation by BIA providing that the expecting differences in FFM and FM associated with age [5] were not verified in the present study (Table 2).

By comparing the correlations obtained using TBW as a size descriptor (Table 3), it can also be seen that a lower dose per kg is required in older patients. This observation is in line with previous literature [7], whereby lower doses of propofol are recommended for older patients. However, the standard bolus dose (1 mg/kg based on TBW) may result in inappropriately low doses in some older patients (Fig. 4d).

The results obtained when TBW is scaled by the corresponding phase angle values showed a stronger correlation level in the Age ≥ 65 group (Fig. 4f). This correlation level was not verified in the Age < 65 group (Fig. 4c). Other authors have previously concluded that BIA derived phase angle can be associated with frailty and that phase angle values can be interpreted as a global marker of health in aging [15]. This fact was noted in the present study, namely on the association observed between standardized phase angle values and CFS scoring in the Age ≥ 65 group (Fig. 2). Therefore, the differences between the correlation levels obtained for the scalar TBW•θ in both groups appear to be related to the differences in the registered frail scores.

A small number of studies have previously assessed the effect of frailty as an independent PK predictor to determine thiopental [33] and propofol [34] dose. Additionally, it has been recommended that perioperative analgesia protocols should be individualized in frail patients [1, 3].

With respect to propofol plasma levels (Table 2), a higher value was found in older patients, thus corroborating the age-related decrease in volume of distribution and inter-compartmental clearance expected in these patients [5, 35]. Higher propofol plasma levels predispose older patients to cardiovascular side effects associated to propofol. In this study, the higher mean MAP variation was observed in the Age ≥ 65 group **(**Table 2**)**. A slow induction has already been recommended for older patients in order to minimize post-induction hypotension [7]. Thus, when infusion rates higher than those adopted in this study are considered, increased MAP variations may be expected. Consequently, since low MAP and deep hypnosis is a predictor of excessive hospital length of stay and post-operative mortality [36], slow titration and monitoring of propofol action is judicious in this group of patients during induction of general anesthesia.

There are potential limitations associated to this study specifically the delay between clinical LOC and blood sample collection. Nevertheless, the average time was not significantly different between groups and the range of plasma concentrations measured ascertained the differences observed in propofol dose to LOC and hemodynamic side effects. Additionally, in this study, each patient received a standardized dose of fentanyl or remifentanil, according to the scheduled surgery. Notwithstanding the synergistic interaction between opioids and propofol for the suppression of purposeful movement to skin incision, the effect of opioids in the absence of a painful stimulus is limited [37]. It was shown by Milne et al. [38] that low target remifentanil concentrations (< 4 ng/mL) have low hypnotic potency and therefore a limited impact on propofol dose requirements when a painful stimuli is not applied. With respect to fentanyl, it has been concluded that the interaction between fentanyl and propofol for LOC is minor [39, 40].

## Conclusion

For the fixed infusion rate of propofol (2000 mg/h) used in the present study, a lower dose of propofol based on TBW is suitable in older patients when compared to that required for adult patients. Nevertheless, the cumulative declines of physiologic systems and the baseline vulnerability to adverse outcomes specific of frail patients should also be measured to estimate individualized dosage profiles. Determination of phase angle appears to be an easy and reliable tool to assess frailty in older patients. A multivariate analysis comparing adult and older healthy patients with adult and older frail patients is necessary to accurately determine the effect of frailty on propofol induction dose.

## Data Availability

The datasets used and/or analyzed during the current study available from the corresponding author on reasonable request.

## Abbreviations

ASA: American Society of Anesthesiology
BCM: Body composition monitor
BIA: Bioelectrical impedance analysis
BIS: Bispectral index
BMI: Body mass index
CFS: Clinical frailty scale
FFM: Fat free mass
FM: Fat mass
GC/IT-MS: Gas chromatography/ion trap-mass spectrometry
LBW: Lean body weight
LOC: Loss of consciousness
MAP: Mean arterial pressure
STROBE: Strengthening the reporting of Observational studies in epidemiology
TBW: Total body weight
